# Enhanced *in vitro* angiogenic behaviour of human umbilical vein endothelial cells on thermally oxidized TiO_2_ nanofibrous surfaces

**DOI:** 10.1038/srep21828

**Published:** 2016-02-17

**Authors:** Ai Wen Tan, Ling Ling Liau, Kien Hui Chua, Roslina Ahmad, Sheikh Ali Akbar, Belinda Pingguan-Murphy

**Affiliations:** 1 Department of Biomedical Engineering, University of Malaya, 50603 Kuala Lumpur, Malaysia; 2 Department of Physiology, Faculty of Medicine, Universiti Kebangsaan Malaysia, 50300 Kuala Lumpur, Malaysia; 3 Department of Mechanical Engineering, University of Malaya, 50603 Kuala Lumpur, Malaysia; 4 Department of Materials Science and Engineering, The Ohio State University, Columbus, OH 43210, USA

## Abstract

One of the major challenges in bone grafting is the lack of sufficient bone vascularization. A rapid and stable bone vascularization at an early stage of implantation is essential for optimal functioning of the bone graft. To address this, the ability of *in situ* TiO_2_ nanofibrous surfaces fabricated via thermal oxidation method to enhance the angiogenic potential of human umbilical vein endothelial cells (HUVECs) was investigated. The cellular responses of HUVECs on TiO_2_ nanofibrous surfaces were studied through cell adhesion, cell proliferation, capillary-like tube formation, growth factors secretion (VEGF and BFGF), and angiogenic-endogenic-associated gene (VEGF, VEGFR2, BFGF, PGF, HGF, Ang-1, VWF, PECAM-1 and ENOS) expression analysis after 2 weeks of cell seeding. Our results show that TiO_2_ nanofibrous surfaces significantly enhanced adhesion, proliferation, formation of capillary-like tube networks and growth factors secretion of HUVECs, as well as leading to higher expression level of all angiogenic-endogenic-associated genes, in comparison to unmodified control surfaces. These beneficial effects suggest the potential use of such surface nanostructures to be utilized as an advantageous interface for bone grafts as they can promote angiogenesis, which improves bone vascularization.

The success rate of implantation and bio-integration of a bone graft into the body is dependent on the outcome of numerous complex events such as bone healing, bone regeneration and bone formation, in which multiple interactions between the implant surface and different types of cells and tissues are involved^1^,^2^. Since the extent of the bone-implant integration is profoundly influenced by the surface properties of the implant, various surface modification techniques have been explored to improve the bone-implant interaction in order to achieve better clinical performance^3^,^4^,^5^,^6^,^7^. Bone is a highly vascularized tissue that contains multiple cell types, including osteogenic cells and endothelial cells^8^,^9^. However, most of these existing works focused mainly on the role and function of the bone-forming osteoblasts to enhance osteo-integration with the surface of the implant, rather than upon the role of vessel-forming endothelial cells, which also play a prominent role in bone vascularization. Nonetheless, several studies have demonstrated that the clinical success of the bone grafts is reliant not only on osteo-integration but also on bone vascularization^10^,^11^,^12^. It has been reported that a rapid and stable bone vascularization at the early stage of implantation is required for optimal functioning of a bone graft^10^,^11^,^12^. Therefore, bone vascularization has been recognized as one of the key contributors to the process of osteogenesis, specifically during bone healing after an implantation procedure^10^,^11^,^12^.

Upon insertion of a bone implant, an acute inflammatory response occurs, followed by a series of repair processes which initiate bone healing^1^,^13^. Bone healing requires active endothelial proliferation, migration and differentiation in order to form new blood vessels or microvasculature from pre-existing blood vessels, a process known as angiogenesis^14^. New blood vessels are essential in supplying oxygen and nutrients, and recruiting precursors or inflammatory cells to the injury site of the bone, as well as removing waste products^15^,^16^. They also play an important role in the maintenance of homeostasis by providing a communicative network between the bone and adjacent tissues^12^,^16^. Several studies have shown that angiogenesis occurs predominantly before the onset of osteogenesis, and these newly formed blood vessels ensure steady transport of osteoblast precursors to the remodelling sites^9^,^16^. In addition, studies have also demonstrated that defective angiogenesis or insufficient bone vascularization in the initial phase after bone implantation results in inadequate oxygen supply, which causes hypoxia and cellular necrosis. Consequently, this leads to the formation of fibrous tissue around the implant which ultimately causes impaired bone healing and poor implant integration^17^,^18^. Since angiogenesis is a prerequisite process for successful osteo-integration during bone healing, an implant surface that can support the growth of endothelial cells can potentially improve the success rate of a bone graft implantation, and the current paper addresses this^9^,^19^.

Titanium (Ti) and its alloys have been widely employed as bone graft materials in numerous clinical devices, including bone and joint replacement, dental implants, prostheses, cardiovascular implants and maxillofacial and craniofacial treatments^20^,^21^. The main factors contributing to their widespread use in the field of biomedical implantations include their favourable mechanical properties, high corrosion resistance, and superior biocompatibility^22^. Their superior biocompatibility is mainly attributed to a thin layer of titanium dioxide (TiO_2_) that forms spontaneously on their surface when exposed to atmospheric conditions. Since the surface topography of the bone implant plays a key role in modulating cellular response, surface modification of Ti based substrate in generating various TiO_2_ nanofeatures has been regarded as one of the most promising approaches to promote better osteo-integration. Particularly, TiO_2_ nanofibers (NFs) have been considered as the most preferred substrate surface for implantable devices due to their high aspect ratio and morphological similarity to natural extra cellular matrix (ECM)^23^. The aspect ratio and physical shape of these NFs were reported to resemble the needle-like shape of crystalline hydroxyapatite (HA) and collagen fibers found in the bone, providing a microenvironment with physical cues that are conducive to cellular organization, survival and functionality^24^,^25^.

To date, the fabrication of TiO_2_ NFs/NWs has been accomplished using electrospinning, anodization, hydrothermal treatment, and laser ablation^21^. However, there are several problems associated with these methods, such as the problem of phase purity, crystallinity and incorporation of impurity^26^. Further post-treatments have to be performed in order to obtain a pure phase structure, and these procedures are neither fast nor cost-effective. Instead of using such expensive and complex methods, we have adopted an inexpensive process originally developed at Ohio State University that grows TiO_2_ NFs directly from a titanium alloy substrate via thermal oxidation under an oxygen deficient environment with a controlled flow rate^27^,^28^. Additionally, we investigated the *in vitro* clinical feasibility of these as-produced surface nanostructures and observed significant enhancement of cell responses of osteoblasts seeded on the TiO_2_ NFs surfaces, compared to the control counterpart^29^. Given the importance of angiogenesis in stimulating osteogenesis during bone healing, an implant surface that promotes both processes of osteogenesis and angiogenesis is desirable and expected to achieve optimal osteo-integration. Although the enhanced osteogenic potential of these as-grown TiO_2_ NFs surface structures has been demonstrated in our previous *in vitro* study^29^, the angiogenic potential of these TiO_2_ NF patterned surfaces remains unknown and yet to be determined.

Therefore, the goal of the present study was to evaluate the potential of these thermally oxidized TiO_2_ NFs surface structures for promoting angiogenesis. TiO_2_ NFs were fabricated using the thermal oxidation method as reported previously^29^,^30^,^31^, and the influence of these as-produced TiO_2_ NFs patterned surfaces on the functionality of human umbilical vein endothelial cells (HUVECs) was investigated. In particular, initial cell adhesion, cell proliferation, capillary-like tube formation, growth factors secretion and the gene expression of angiogenic-endogenic-associated factors in HUVECs were examined on the TiO_2_ NFs surfaces in contrast to the unmodified control surfaces after 2 weeks of cell seeding.

## Methods

### Preparation of *in situ* TiO_2_ nanofibers

The thermal oxidation previously reported was used for creating *in situ* TiO_2_ NFs on Ti alloy surfaces^29^,^30^,^31^,^32^. Briefly, Ti-6Al-4V disks (Grade #5, Titan Engineering Pte Ltd, Singapore) with dimension of ø 6.35 × 2 mm were mechanically polished up to #1200 grit, ultrasonically degreased in acetone, methanol and distilled water sequentially, and etched in HCl solution at 80 °C for 10 min to remove any native oxide layer. The discs were then rinsed with distilled water and left to dry in air. The oxidation process was carried out in a horizontal tube furnace (Lindberg, TF55035C) in which the discs were placed at the centre of the furnace and a constant flow of 750 mL/min Argon gas (99.999% purity) was flown into the furnace as the carrier gas. The furnace was heated to 700 °C and held for 8 h before rapid cooling to room temperature. Polished Ti-6Al-4V samples without thermal oxidation were designated as control surfaces and were compared to thermally oxidized samples for all experiments. All the samples were sterilized by autoclaving prior to use in cell culture.

### Isolation and cultivation of HUVECs

This research was conducted with ethical approval from the Universiti Kebangsaan Malaysia Research and Ethical Committee (Reference number: UKM-FF-FRGS0165-2010) and was carried out in accordance with the approved guidelines. Human umbilical vein endothelial cells (HUVECs) were obtained from human umbilical cords of patients who delivered babies at Universiti Kebangsaan Malaysia Medical Centre with informed consent. HUVECs were isolated using 0.1% collagenase type 1 (Worthington, US) and cultured in complete Endothelial Cell Medium (ECM) supplemented with 5% fetal bovine serum (FBS), endothelial cell growth supplement and penicillin/streptomycin (ScienCell, CA) at 37 °C in an incubator of 5% carbon dioxide (CO_2_). The culture medium was changed every 2 days until the cells reached 80–90% confluence. HUVECs at their third passage (P3) were used for this experiment. HUVECs were seeded on the specimens at a density of 30,000 cells/50 μl and allowed to attach for an hour at 37 °C in a 5% CO_2_ atmosphere. Non adherent cells were removed by washing the specimens with phosphate buffer saline (PBS) gently. The specimens were then placed in a 24-well plate and cultured with complete ECM medium. HUVECs seeded on the specimens were harvested after 1, 3, 7, and 14 days of cell culturing for subsequent cell experiments.

### Cell adhesion and morphologies

Field emission scanning electron microscope (FESEM; Zeiss Gemini, Germany) was employed to examine the morphologies of HUVECs seeded on the specimens after the prescribed time period. Briefly, the specimens were washed 3 times in PBS and fixed with 2.4% formalin solution (Sigma Aldrich Co.) for an hour. After fixing, the specimens were washed 3 times again with PBS, placed through a series of graded ethanol dehydrations, and allowed to dry overnight in a freeze dryer (FreeZone, Labconco, USA). Then, the dried specimens were sputter coated with gold and analysed under FESEM.

### Cell proliferation assessment

The quantitative analysis of proliferation of HUVECs was performed using the AlamarBlue assay (Invitrogen, CA) according to the manufacturer’s protocol. After prescribed incubation time, HUVECs were incubated with 10% AlamarBlue reagent for 4 h at 37 °C in a 5% CO_2_ atmosphere for the conversion of resazurin to resorufin. The optical density (OD) was then measured using a microplate reader at 570 nm, with 600 nm set as the reference wavelength. The cell number of each sample was determined according to a linear regression equation derived from the pre-equilibrium standard curve.

### Quantification of VEGF and BFGF protein secretion

The production of VEGF and BFGF proteins was quantified using the Enzyme-linked Immunosorbent Assay (ELISA) Development kit (Peprotech, USA) in accordance to the manufacturer’s recommended protocol. Briefly, HUVECs were cultured in both the sample substrates for the prescribed time period, and proteins from whole cell lysates were collected and quantified using the ELISA kit.

### Matrigel tube formation assay

The assay was conducted by pre-coating a thin layer of 2% agarose gel on a tissue culture dish with three holes created on the agarose gel. The specimens were then placed in the holes created and coated with Growth factor reduced Matrigel^TM^ (BD Biosciences) mixed with 1 × 10^5^ HUVECs suspended in 80 μl medium at a ratio of 1:1. After the Matrigel was allowed to gel in the incubator for 15 min at 37 °C, medium was added to the tissue culture dish. The culture tissue plate was then incubated (37 °C, 5% CO_2_) for 7 days with medium changed every 3 days. At day 7, Matrigel was detached from the specimens and placed on a glass slide for capillary-like tube formation examination under light microscopy (Axiovert S100, Zeiss). The respective images were then captured using digital camera.

### Total RNA extraction, cDNA synthesis and gene expression analysis by Quantitative Real-time Polymerase Chain Reaction (qRT- PCR)

Total RNA was extracted from HUVECs seeded on the samples at day 7 and day 14 using TRI reagent (Molecular Research Center). The procedure was carried out according to the manufacturer’s recommended protocol, which includes homogenization, phase separation, RNA precipitation, RNA wash and RNA solubilization. The RNA precipitation was increased by adding polyacryl carrier (Molecular Research Center). The solubilised RNA extracted was used for the synthesis of cDNA using SuperScript III First-Strand Synthesis SuperMix (Invitrogen). The reaction was carried out according to the protocol recommended by the manufacturer. The protocol conditions were 10 min at 25 ^o^C, 30 min at 50 ^o^C, 5 min at 85 ^o^C and 20 min at 37 ^o^C. The synthesized cDNA was stored at −20 °C and was later used as template to determine the gene expression of angiogenic and endogenic related genes including vascular endothelial growth factor (VEGF), vascular endothelial growth factor receptor 2 (VEGF2), placenta growth factor (PGF), platelet-endothelial cell adhesion molecule 1 (PECAM-1), basic fibroblast growth factor (BFGF), ANGIOPOIETIN-1 (Ang-1), von Willebrand factor (VWF), hepatocyte growth factor (HGF) and endothelial nitric oxide synthase (ENOS) using qRT-PCR. Glyceraldehyde-3-phosphate dehydrogenase (GAPDH) was used as the housekeeping gene to normalize the data. Primers for each gene were designed using Primer 3 software based on published Gen Bank database sequences. The sequences of the primers used are listed in Table 1. The PCR reaction was carried out using Bio-Rad iCycler PCR machine with SYBR green as the indicator. The reaction mixture contained SYBR^®^ Select Master Mix (Applied Biosystem), forward and reverse primers (5 μM each), DNaseRNase free water and 1 μl of cDNA. The reaction conditions were: cycle 1 (1×): Step 1- 50 ^o^C for 2 min; cycle 2 (1×): Step 1- 95 ^o^C for 2 min; cycle 3 (50×): step 1- 95 ^o^C for 10 s, and step 2- 58 ^o^C for 30 s; cycle 4 (1×): step 1- 95 ^o^C for 1 min; cycle 5 (1×): step 1- 55 ^o^C for 1 min; and cycle 6 (70×): step 1- 60 ^o^C to 94.5 ^o^C for 10 s each. The specificity of the primers and PCR protocol were confirmed by the melting curve analysis. The expression level of each gene was then normalized to GAPDH.

### Statistical analysis

All results are expressed as means ± standard error mean (SEM). The results obtained were collected from three different samples. Student’s T-test was utilized to demonstrate the statistical significance between groups, with P < 0.05 was considered to be significant.

## Results and Discussion

### Surface characterization of TiO_2_ NFs

**Figure 1:**
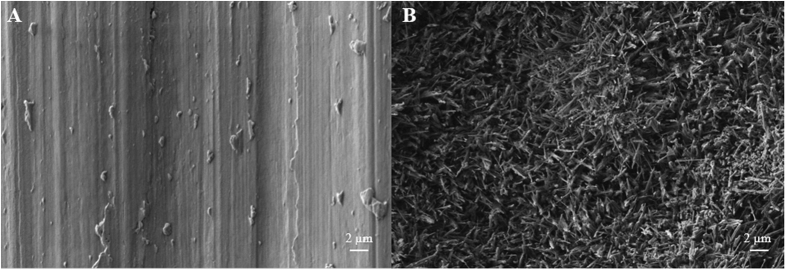
FESEM micrographs of the (**A**) unmodified control surface and (**B**) as-fabricated TiO_2_ NFs surface.

FESEM surface micrographs depicted in Fig. 1 show the unmodified control Ti-6Al-4V surfaces and as-fabricated TiO_2_ NFs surfaces. The nanofibrillar morphological features were evident on the Ti-6Al-4V substrate after the thermal oxidation treatment at 700 °C in Argon ambient for 8 h. The entire surface of the Ti-6Al-4V substrate was covered homogeneously by dense TiO_2_ NFs after the oxidation treatment, typically of dimension about 50 nm in diameter and 785 nm in length. As expected, no nanostructured features were observed on the unmodified control surface. The successful fabrication of these nanomodified TiO_2_ NFs surfaces was ascertained by a detailed surface characterization, which has been published earlier^29^,^31^.

### Morphological observation of HUVECs

**Figure 2:**
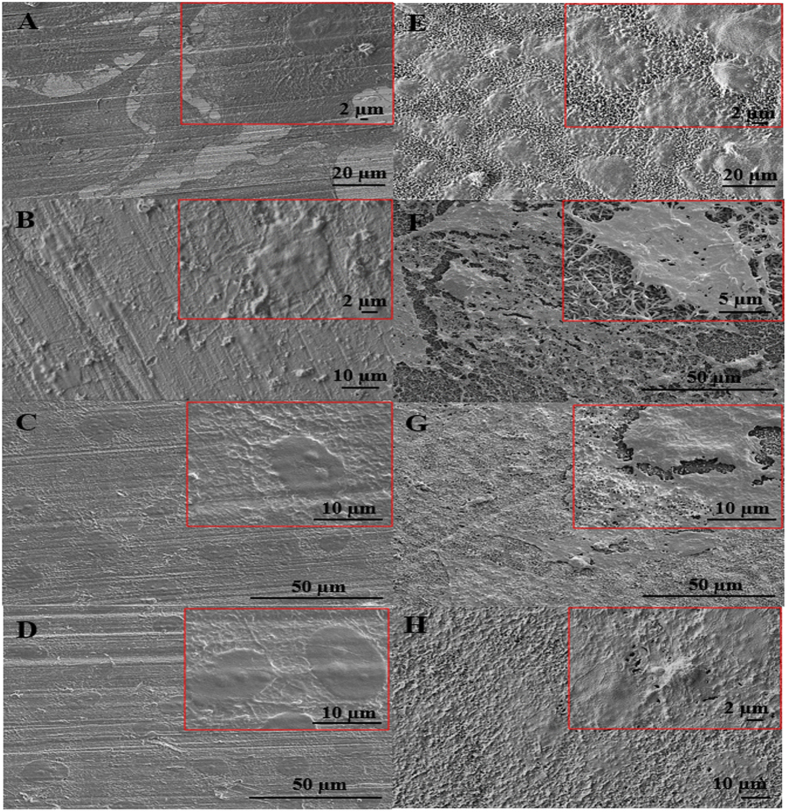
FESEM images show HUVECs adhered on the unmodified control surfaces after (**A**) day 1, (**B**) day 3, (**C**) day 7 and (**D**) day 14 of culture compared to TiO_2_ NFs surfaces after (**E**) day 1, (**F**) day 3, (**G**) day 7 and (**H**) day 14 of culture. The images in the right inset are shown in higher magnification.

To determine how these surface nanofeatures affect the morphology of HUVECs, HUVECs were grown on nanomodified TiO_2_ NFs surfaces and unmodified control sample surfaces, respectively. Their morphological responses to both the surfaces were investigated through FESEM after 2 weeks of culture as shown in Fig. 2. Our results show that HUVECs were able to spread and adhere on both control and NFs surfaces, and significant morphological differences over time were observed between these groups. On the unmodified control surface, HUVECs were flattened and were partly spread out after cultured for one day (Fig. 2A). The spreading and numbers of HUVECs were only slightly improved after day 3, 7 and 14 days of culture, as shown in Fig. 2B–D respectively. On the contrary, HUVECs adhered and spread well on the NFs surfaces after cultured for one day, and the cells exhibited a cobblestone-like morphology (Fig. 2E). At day 3, pronounced protrusion of filopodia from HUVECs attaching tightly to the underlying NFs surfaces and covering a vast area of the surfaces was observed, as shown in Fig. 2F, indicating a good spreading morphology, and enhanced cell-cell communication and cell-substrate adhesion. The cells extended further by stretching out their filopodia toward each other to form an intercellular connection which nearly reached a continuous cell layer on day 7 (Fig. 2G), with a confluent cell monolayer developed by day 14 (Fig. 2H).

Cell adhesion is the first interaction when cells come into contact with a material *in vitro*. Cells can only continue to grow and differentiate if they can adhere to the surface of implant and, thus, cell adhesion is important in defining the quality of cell-implant interfaces. In aggregates, our results show that TiO_2_ NFs surfaces can promote better cell adhesion and spreading than the unmodified control sample surfaces. The as-grown NFs surfaces have an advantage of providing a larger surface area for the cells to attach and their irregular nanofibrillar structures act as the cues for the cells to anchor to, contributing to a lock-in cell configuration and thereby enhanced the cell-cell and cell-substrate adhesions^33^. Therefore, a greater number of cellular interconnections and a higher number of anchored cellular filopodia were evidenced on the TiO_2_ NFs surfaces. Conversely, the unmodified control surface has a much lower surface area and does not render much topographical cues, and hence could not provide adequate anchorage sites for the cells to attach, as indicated by Fig. 2A–D, which show filopodia that are not particularly pronounced even after 14 days of cell culturing.

It is widely accepted that biological response between the implant surface and the cells in contact is closely associated with the surface wettability, surface roughness and crystallinity^34^,^35^. Various studies have shown that implant surface possessing a higher degree of surface wettability, surface roughness and crystallinity are more effective in promoting initial protein adsorption onto the surface, and thereby enhance cell attachment^7^,^36^,^37^. Therefore, in view of our results, another possible explanation for the increased HUVECs adhesion on the TiO_2_ NFs surfaces is that the as-grown NFs possess increased surface roughness and surface wettability, as well as greater degree of crystallinity, both of which have been proven in our previous studies^29^,^31^.

### Proliferation of HUVECs

**Figure 3:**
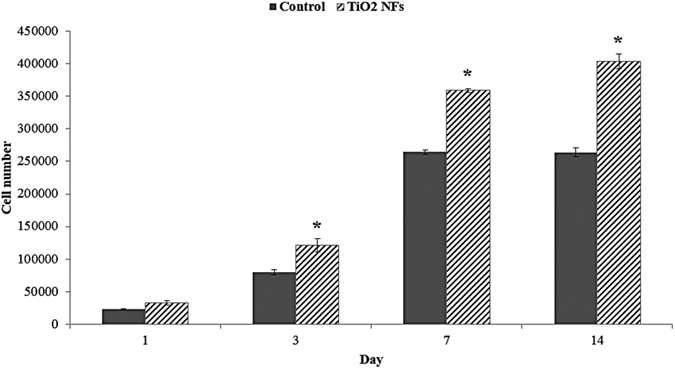
Cell proliferation of HUVECs seeded on TiO_2_ NFs surface in comparison to the unmodified control surface at days 1, 3, 7 and 14. Statistical significance was assessed relative to the control surface for each day (*P < 0.05).

Proliferation of endothelial cells is an important process in the formation of new blood vessels^38^. Therefore, the effect of the TiO_2_ NFs surfaces on proliferation of HUVECs was investigated using the AlamarBlue assay, which measures the amount of oxido-reduction reactions in the cells. The cell proliferation results for both control and TiO_2_ NFs surfaces after 2 weeks of cell culturing are shown in Fig. 3. As indicated, HUVECs showed a progressive growth with time, from day 1 to day 14, for TiO_2_ NFs surfaces. For the case of control surface, the growth of HUVECs reached its maximum at day 7 and then maintained a plateau up to day 14. This observation is likely due to the limited cellular growth space, as the unmodified control sample surface has a much lower surface area than the NFs surface. Noticeably, at each time point, a significantly higher number of HUVECs were found on the thermally oxidized TiO_2_ NFs surface as compared to the control surface, except for day 1, in which there was no significant difference between these groups (P < 0.05). In conjunction with the cell adhesion results obtained via FESEM, our results showed that the as-grown nanofibrillar structures are more beneficial in terms of promoting cell adhesion and proliferation of HUVECs, than the unmodified control surfaces.

### Measurement of VEGF and BFGF protein secretion

**Figure 4:**
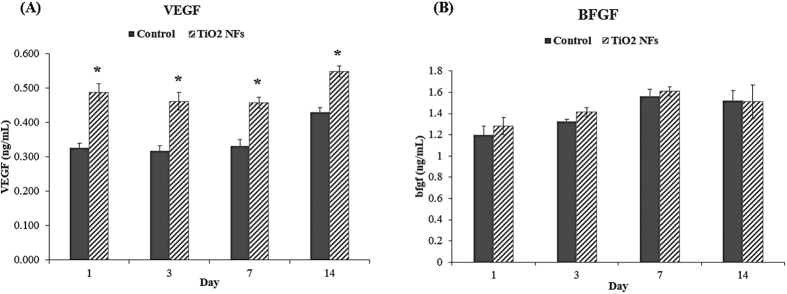
(**A**) VEGF and (**B**) BFGF proteins production level of HUVECs cultured on TiO_2_ NFs surfaces in comparison to the unmodified control surfaces at days 1, 3, 7 and 14. Statistical significance was assessed relative to the control surfaces for each day (*P < 0.05).

The secretion concentration of the VEGF and BFGF proteins over 2 weeks of cell culture were determined quantitatively using ELISA assay, which is a widely used highly sensitive method in detecting low level of cytokines due to their biological catalysis and specificity^39^. These concentrations of secreted VEGF and BFGF proteins were detectable in both sample surfaces, as shown in Fig. 4. For VEGF protein secretion, it was observed that at each time point, the level of VEGF protein was found to be significantly higher on the NFs surfaces than that on the unmodified control surface (P < 0.05). However, for BFGF protein secretion, there were no statistically significant differences in the level of BFGF protein between the two sample surfaces, even though the concentration on the NFs surfaces seemed to be slightly higher than the unmodified control surface at each time interval.

VEGF and BFGF are the most potent and widely investigated pro-angiogenic growth factors^40^. VEGF is known to be a major angiogenic modulator involved in initiating the signalling cascade during vascularization in endothelial cells^41^. VEGF promotes the formation of blood vessels by stimulating endothelial proliferation, migration and capillary tube formation^17^. BFGF induces angiogenesis through functioning as an autocrine and paracrine factor, which stimulates proliferation and migration of endothelial cells^15^. Our results showed that HUVECs cultured on NFs surfaces produced higher secreted levels of VEGF and BFGF proteins than those cells cultured on the unmodified control surface. Since both the VEGF and BFGF have been implicated as regulators of angiogenesis, the higher secreted levels of these proteins implies enhanced angiogenic activity of HUVECs cultured on the sample surfaces. On this basis, we can provisionally conclude that NFs surfaces enhance the ability of HUVECs in producing angiogenic factors *in vitro*.

### Observation of capillary-like tube formation

**Figure 5:**
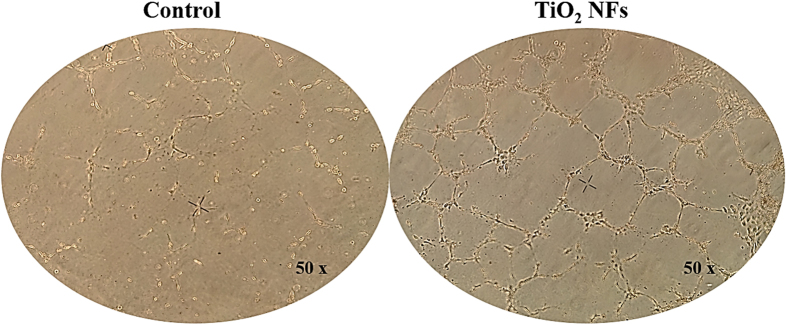
Digital images of capillary network formation on Matrigel by HUVECs seeded on both the unmodified control surfaces and TiO_2_ NFs surfaces after 7 days of culture.

The angiogenic capability of HUVECs seeded on the different sample surfaces was assessed using an *in vitro* capillary-like tube formation assay on Matrigel after 7 days of incubation. A hallmark of endothelial cells is their ability to undergo morphogenic changes to form a network of tubular structures and thus the formation of capillary-like tubes has been reported as a marker of endothelial cell differentiation^1^,^42^. As evidenced by Fig. 5, the formation of capillary-like tube network by HUVECs seeded on TiO_2_ NFs surfaces was enhanced as compared to those on the unmodified control surfaces. Prominent networks of capillary-like tubes with higher number of branching points and longer capillary tube lengths were observed on the TiO_2_ NFs surfaces, implying that TiO_2_ NFs surfaces possess a greater angiogenic ability in inducing greater endothelial cell differentiation. A likely explanation for this observation is that the as-grown nanofibrous surfaces contain more topological cues and provide more attachment sites for the cells to contact and adhere to, thereby increasing the cell-cell contacts and formation of tube-like structures.

### Expression of angiogenic-endogenic-related genes

**Figure 6:**
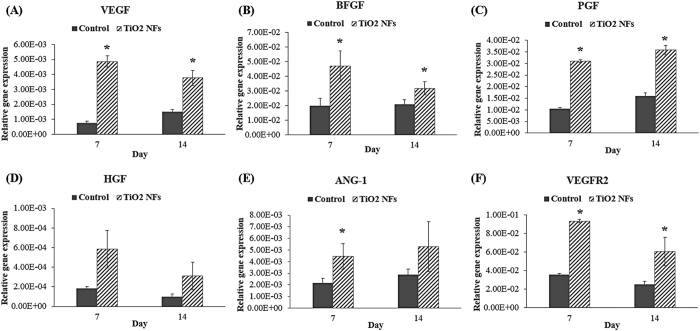
Relative expression of angiogenic-associated genes: (**A**) VEGF, (**B**) BFGF, (**C**). PGF, (**D**) HGF, (**E**) Ang-1 and (**F**) VEGFR2, by HUVECs cultured on both the unmodified control surfaces and TiO_2_ NFs surfaces for 7 days and 14 days. Statistical significance was assessed relative to the control surfaces for each time interval (*P < 0.05).

**Figure 7:**
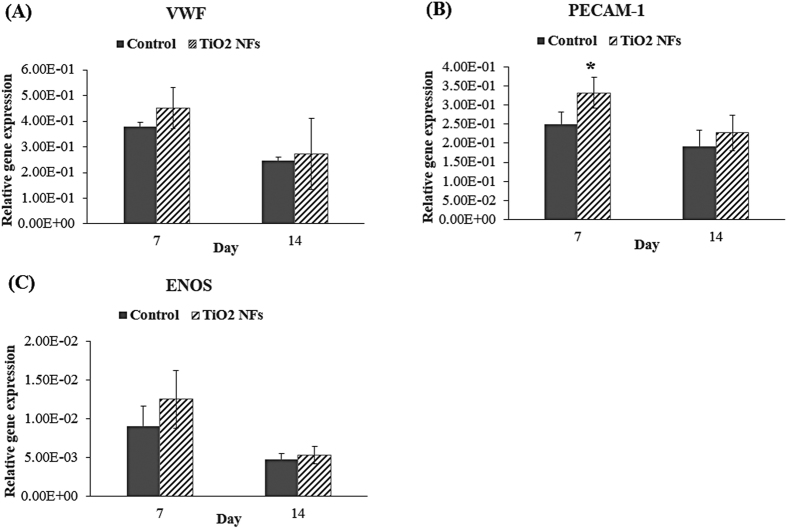
Relative expression of endogenic-associated genes: (**A**) VWF, (**B**) PECAM-1 and (**C**) ENOS, by HUVECs cultured on both the unmodified control surfaces and TiO_2_ NFs surfaces for 7 days and 14 days. Statistical significance was assessed relative to the control surfaces for each time interval (*P < 0.05).

To further examine the influence of the as-grown TiO_2_ NFs surface structures on the differentiation of HUVECs at the molecular level, the mRNA expressions of angiogenic-endogenic-associated genes, including VEGF, BFGF, PGF, HGF, Ang-1, VEGFR2, PECAM-1, VWF, and ENOS, were characterized quantitatively by real-time PCR after cultured for 7 and 14 days on both the sample surfaces, and the results are depicted in Fig. 6 and 7, respectively. As illustrated in Fig. 6 and 7, our data clearly show that the expression levels of VEGF, BFGF, PGF and VEGFR2 on NFs surfaces were significantly higher than that of the unmodified control surface at each time intervals (P < 0.05). Although the expression levels of Ang-1 and PECAM-1 on the as-grown NFs surfaces were higher than the unmodified control surface, the significant differences between these groups were only detected at day 7 (P < 0.05). Meanwhile, no significant differences were found among these groups for the expression levels of HGF, VWF and ENOS, even though the expression levels on NFs surfaces seem to be higher than the unmodified control surfaces at each incubation time. However, for VEGF, a variation between the results of ELISA (Fig. 4) and the results of mRNA expression on the NFs surfaces was observed. NFs surfaces displayed higher content of VEGF protein at day 14 compared to day 7 (Fig. 4), which is different from the trend of mRNA expression profile of VEGF (Fig. 6). This discrepancy may be due to a delayed variation in protein level of VEGF relative to its mRNA level with incubation time, since protein is a downstream product of gene^43^,^44^. Moreover, except for PGF and Ang-1, the expression levels of all the angiogenic-endogenic-associated genes were found to be peaked at day 7 on the NFs surfaces, suggesting that these surface nanostructures can promote HUVECs progressing into a more mature phenotype at an earlier time compared to the unmodified control counterpart.

The angiogenic-associated genes of interest in this study were VEGF, VEGFR2, PGF, HGF, BFGF and Ang-1. VEGF and its corresponding receptor (VEGFR2), PGF, HGF and BFGF have been well characterized as the major pro-angiogenic growth factors and are known to regulate angiogenesis by promoting endothelial cell proliferation, migration, and tube forming activity^13^,^40^,^45^. As exemplified in Fig. 6, the expressions of the angiogenic-associated genes were strongly expressed on the NFs surfaces than on the unmodified control surfaces, implying a better angiogenic potential of the as-grown NFs over the corresponding control counterpart. Ang-1, a member of the angiopoietin family of growth factor, has been identified as the ligand of the endothelial cell-specific Tie2 receptor with important role in vascular development and angiogenesis^41^. It is known to control the later stage of blood vessel formation, such as stabilizing mature blood vessels by promoting an interaction between endothelial cells and their pericytes^46^. It is reported that a stabilization process is important to provide the structural support to the blood vessel after the formation of a vascular structure^40^. High expression of Ang-1 in HUVECs cultured on NFs surfaces suggest that these as-grown surface nanostructures play a better role in assisting the stabilization of mature blood vessel.

We also investigated the mRNA expression of the endogenic-associated genes, including VWF, PECAM-1 and ENOS. The presence of all these genes commonly found in endothelial cells and thus, they are implicated as typical markers for endothelial cells. VWF is a blood clotting protein that is involved in angiogenesis and bone healing. It is produced uniquely by functional endothelial cells and thus it has been regarded as the functional marker for mature endothelial cells^45^. It also plays an important role in the maintenance of homeostasis by mediating the adhesion of platelets to the vascular wall at the site of injury^38^. As revealed in Fig. 7, position expression of VWF gene expression on the modified NFs surfaces over unmodified control surfaces suggest that HUVECs had gained some endothelial functions and characteristics after growing on the nanofibrous surfaces. The expression of PECAM-1 by HUVECs is known to be crucial for vessel formation and maintenance^9^. PECAM-1 is a cell adhesion molecule that is expressed on platelet and at endothelial cell intercellular junctions and it plays a role in endothelial cell-cell adherence and migration^47^. As evidenced from Fig. 7, PECAM-1 expression on NFs surfaces was generally higher than that of the unmodified control surface, indicating increased endothelial cell proliferation and migration on the NFs surfaces, which is in good agreement with our cell proliferation results as shown in Fig. 3. ENOS is a constitutively expressed gene in endothelium that produces basal nitric oxide (NO) and has a critical role in regulating endothelial cell growth and angiogenesis^47^. Although no significant difference was detected between these groups, the expression of ENOS on NFs surfaces was generally higher than the unmodified control surface, demonstrating the possibility that the as-grown NFs surfaces are preferred by HUVECs in promoting angiogenesis. Taken together, the expressions of angiogenic-endogenic genes of HUVEVs were positively expressed by the NFs surface structures fabricated using thermal oxidation in the present study and thus, we can draw a conclusion that NFs surface structures can provide a microenvironment that is more favourable for HUVECs in promoting angiogenesis.

## Conclusions

In the present study, we examined the potential of the as-grown thermally oxidized TiO_2_ NFs surface structures to promote angiogenesis by evaluating the *in vitro* cellular response of HUVECs on the resulting surfaces after 2 weeks of culture. Our results indicate that these thermally oxidized nanostructures of titania interact more efficiently with HUVECs in expediting angiogenesis. Significant enhancement of cell adhesion, cell proliferation and cell differentiation of HUVECs on the TiO_2_ NFs surfaces were observed as compared to those on the unmodified control surfaces. These as-grown nanofibrillar surface structures were also found to significantly enhance functional properties such as VEGF and BFGF protein secretion by HUVECs. Furthermore, the levels of all angiogenic-endogenic-associated genes were positively expressed by the HUVECs cultured on TiO_2_ NFs surfaces, as compared to the unmodified control surfaces. Taken together, our results provide convincing evidence on the effectiveness of the as-produced TiO_2_ NFs surfaces in terms of promoting angiogenesis. Considering the enhanced osteogenic potential of these surface structures based on our previous *in vitro* study, we suggest that these TiO_2_ NFs surfaces can be considered as an advantageous interface for a bone graft, as they simultaneously promote osteogenesis and angiogenesis, which are equally crucial to achieve an optimal bone-implant integration.

## Additional Information

**How to cite this article**: Tan, A. W. *et al.* Enhanced *in vitro* angiogenic behaviour of human umbilical vein endothelial cells on thermally oxidized TiO_2_ nanofibrous surfaces. *Sci. Rep.* **6**, 21828; doi: 10.1038/srep21828 (2016).

## Figures and Tables

**Table 1:** Description of primers used in qRT-PCR for gene expression analyses.

GENE	ACCESSION NO.	PRIMER 5′-3′	PRODUCT SIZE
GAPDH	NM_002046	F-5′-TCC CTG AGC TGA ACG GGA AG-3′R:5′GGA GGA GTG GGT GTC GTC GCT GT-3′	217
PGF	NM_002632	F-5′-GTT CAG CCC ATC CTG TGT CT-3′R-5′-CTT CAT CTT CTC CCG CAG AG-3′	199
VEGF	NM_001033756	F-5′-CCC ACT GAG GAG TCC AAC AT-3′R-5′-AAA TGC TTT CTC CGC TCT GA-3′	173
VEGFR2	NM_002253	F-5′-GCA ATC CCT GTG GAT CTG AA-3′R-5′-ACT CCA TGC CCT TAG CCA CT-3′	193
PECAM-1	NM_000442	F-5′-TCA AAT GAT CCT GCG GTA TTC-3′R-5′-CCA CCA CCT TAC TTG ACA GGA-3′	169
BFGF	NM_002006	F-5′-CCG TTA CCT GGC TAT GAA GG-3′R-5′-ACT GCC CAG TTC GTT TCA GT-3′	158
ANGIOPOIETIN-1	NM_001146	F-5′-GAA GGG AAC CGA GCC TAT TC-3′R-5′- GCT CTG TTT TCC TGC TGT CC-3′	108
VWF	NM_000552	F-5′-GAC CTT GCT GAA GAG TCA TCG-3′R-5′-GCC AGT CAG CTT GAA ATT CTG-3′	184
HGF	NM_001010932	F-5′-CTG GTT CCC CTT CAA TAG CA-3′R-5′- CTC CAG GGC TGA CAT TTG AT-3′	168
ENOS	NM_000603	F-5′-CTC CAG CCC CGG TAC TAC TC-3′R-5′-TTA GCC ACG TGG AGC AGA CT-3′	139
